# Harnessing clonal diversity in grapevine: from genomic insights to modern breeding applications

**DOI:** 10.1007/s00122-025-04986-w

**Published:** 2025-08-04

**Authors:** Paolo Callipo, Maximilian Schmidt, Timo Strack, Hannah Robinson, Akshaya Vasudevan, Kai P. Voss-Fels

**Affiliations:** Department of Plant Breeding, Hochschule Geisenheim University, Geisenheim, Germany

## Abstract

Grapevine has been clonally propagated for thousands of years. Though clonal propagation aims at maintaining varietal identity, somatic mutations and epigenetic modifications accumulated over hundreds to thousands of years lead to intra-varietal diversity. This intra-varietal variation is a very valuable resource in grapevine breeding, as it creates the opportunity to improve important traits related to yield, phenology, stress tolerance, and quality without altering the varietal identity which is extremely important for the industry. Recent advances in genomics, epigenetics, and phenotyping technologies are providing completely new opportunities to gain functional insights into the drivers underlying trait variation and to explore this for accelerated grapevine breeding. This review discusses the interaction between somatic mutations, epigenetic regulation, and emerging breeding technologies. We begin by exploring the phenotypic variation observed within clonal populations across various commercially important varieties, focusing on both agronomic and winemaking-related traits. Next, we examine the extent of genomic and epigenomic variation among clones, highlighting known mutations responsible for somatic variants. We also address how grapevine clonal populations serve as an advantageous model for understanding how genetic and epigenetic variants shape complex trait variation. Given recent advances, we discuss the potential of predictive breeding strategies to accelerate clonal evaluation and how genome editing technologies open new opportunities for targeted genetic improvements without passing through the tedium and unpredictability of clonal selection, driven by natural mutation. Ultimately, these new breeding technologies enable the integration of advanced methods into breeding programmes, optimizing grapevine performance while preserving the unique heritage of historic cultivars.

## Clonal diversity and its role in grapevine evolution and breeding

Grapevine (*Vitis vinifera* L.) has been cultivated since the advent of agriculture (Dong et al. 2023), is now grown worldwide, and ranks as the third most valuable horticultural crop, with a farm gate value of 68 billion US dollars in 2016 (Alston and Sambucci 2019). It is propagated vegetatively, in order to preserve the genome and the phenotypic traits that are forming to the identity of the major cultivated grapevine varieties (This et al. 2006). Some important cultivars, like Pinot noir, belong to lineages with a long history. Archaeobotanical evidence indicates that genotypes similar to modern Pinot noir were cultivated and that vegetative propagation was extensively practiced during Roman times (Bouby et al. 2013). Even if the goal is to preserve the genetic integrity of a cultivar, this is not entirely achievable over centuries, as the genome of each clone is continuously reshaped over time by somatic mutations, which are genetic changes occurring in cells outside of a strictly segregated germline (Vondras et al. 2019) and by epigenetic modifications, such as DNA methylation and histone modification (Douhovnikoff and Dodd 2015).

**Fig. 1:**
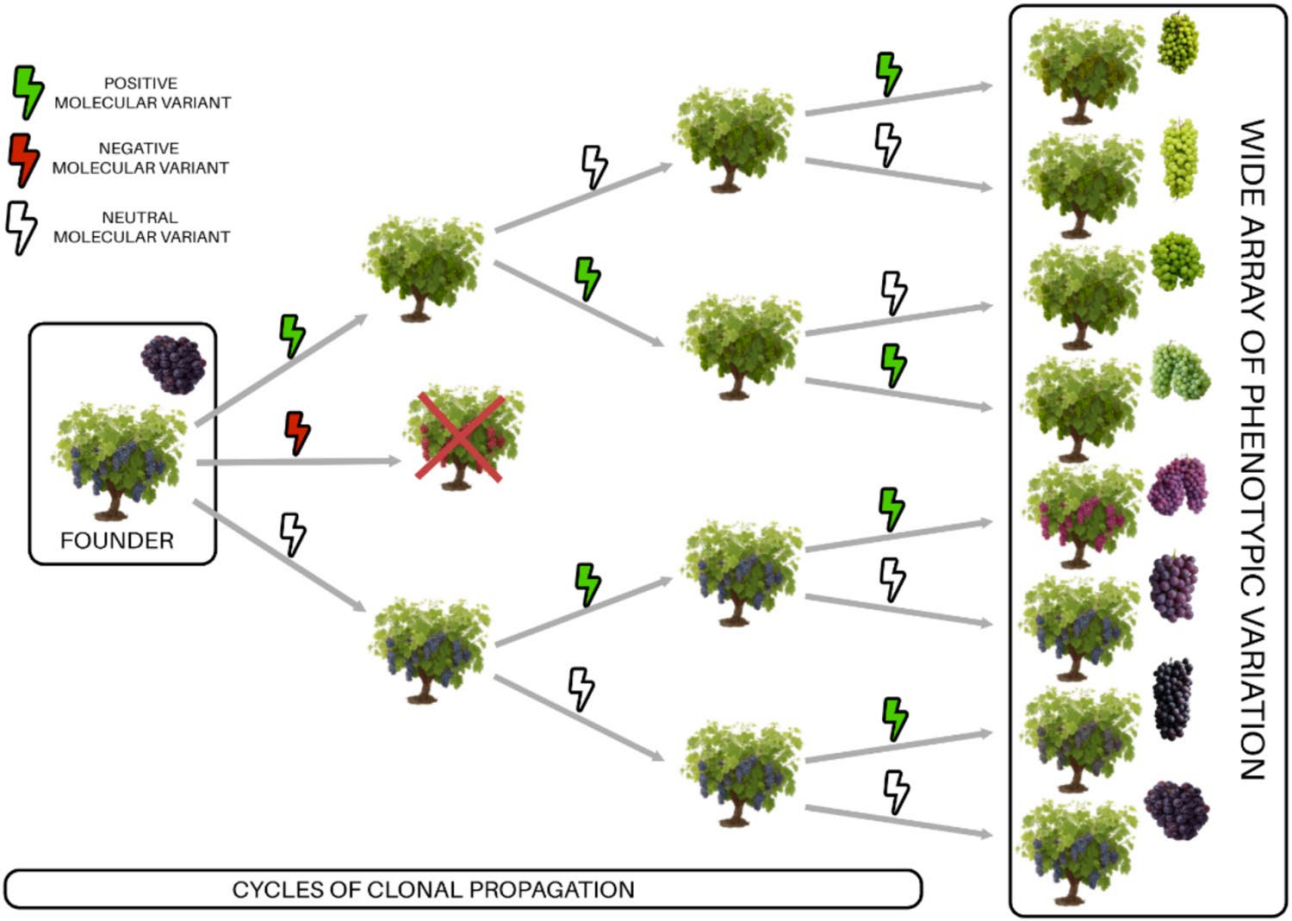
Schematic representation of how repeated cycles of clonal propagation in grapevine can generate diverse clonal lineages. Note that vegetative propagation of many important cultivars, such as Pinot noir or White Riesling, has been practiced over hundreds to thousands of years of propagation cycles. Green symbols represent beneficial molecular variants, while red symbols indicate negative ones. Crossed-out vines are not propagated as part of maintenance selection to preserve desirable traits. Molecular variants can arise through both genetic mutations and epigenetic modifications, contributing to the diversity observed in clonally propagated grapevine cultivars

Through these molecular variants, new genotypes, and sometimes even new phenotypes, can arise. In fact, it is common to find clones with noticeable phenotypic differences within the same variety. However, as long as these variations remain within an acceptable range for trait characteristics strictly defined in varietal catalogues, they are still considered a clone of that single variety (Pelsy 2010). Viticulturists and breeders have played a key role in this process by monitoring vineyards for natural variations. They have selected against unwanted changes to maintain cultivar traits, or selected beneficial variants to develop new clones. When a new phenotype emerges, if it is visually appealing or agronomically advantageous, for instance, displaying stable yield or low-maintenance canopy traits, it is selected and conserved through vegetative propagation (Fig. 1). Older cultivars, with their longer histories of propagation, tend to exhibit greater variation than more modern ones. For example, the ancient cultivar Pinot noir that comprises several hundred distinct clones is assumed to have been vegetatively propagated for over 2000 years (Jackson 2000).

These wide range of clones serves as a valuable reservoir of genetic variation for grapevine improvement. Through clonal selection, it is possible to refine traits while staying within legally acceptable boundaries for trait variations and thereby preserving the variety’s essential characteristics and most importantly, the variety name (Jackson 2000). In many traditional winemaking regions, clonal diversity is emerging as a key adaptive strategy to tackle challenges posed by climate change (Tortosa et al. 2020; van Houten et al. 2020; Duchêne 2016). These regions rely on a few historic grapevine cultivars with an enormous commercial value and cultural significance that makes any attempt to substitute them with modern varieties exceptionally challenging. Instead, the process of selecting clones and harnessing natural somatic mutations have the potential to both meet the markets demand for wine obtained from historical cultivars and improve traits that can facilitate viticulture under climate change.

Clonal selection in grapevine began in Europe in the early nineteenth century and is considered a very important tool in grapevine breeding. It has been fundamental for propagating virus-free planting material and later for selecting superior clones with improved desired traits (Mannini 2000; International Organisation of Vine and Wine 2017). In a grapevine clonal selection program, breeders capitalize on naturally occurring genetic variation, relying on (i) spontaneous mutations or induced mutagenesis that cause desired trait changes and (ii) the ability to reliably detect them among thousands to millions of vines, without making deliberate crosses. During the last century, different grapevine breeding programmes have exploited this diversity and successful examples of clonal selection efforts can be found in many grape growing countries such as Germany (Ruehl et al. 2015), France (Lacombe and Audeguin 2008), Spain (Ibáñez et al. 2015), Portugal (Gonçalves et al. 2019), Hungary (Farkas et al. 2023), and Slovenia (Rusjan et al. 2024). Similar efforts are undertaken in other major viticultural nations.

**Fig. 2:**
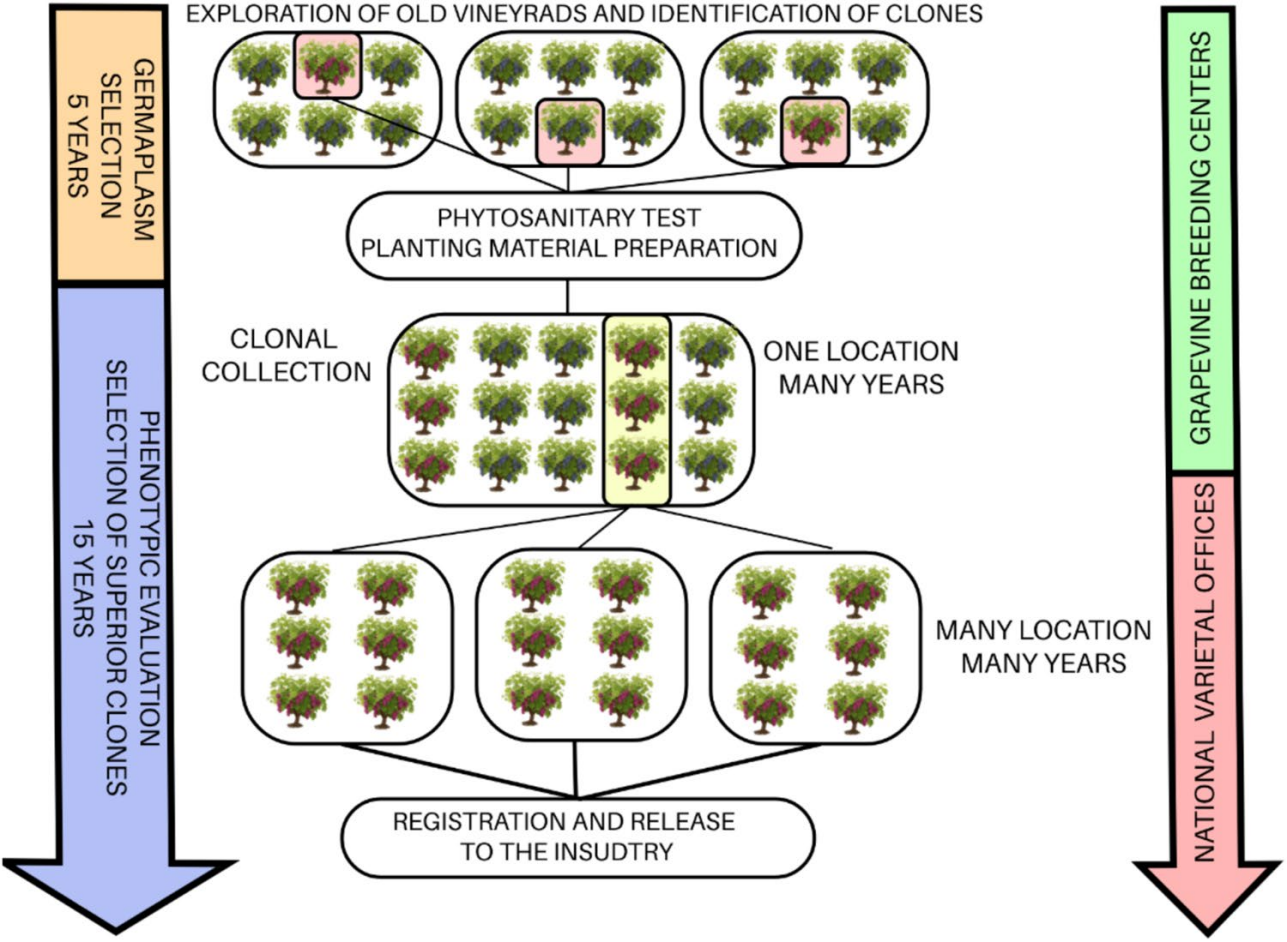
Schematic overview of the grapevine clonal selection pipeline, from initial germplasm exploration to commercial release. The diagram illustrates two main phases: germplasm selection (approximately 5 years) and phenotypic evaluation and selection of superior clones (up to 15 years). The process begins with exploration and identification of promising grapevine clones from old vineyards. Selected clones undergo phytosanitary checks and are subsequently propagated in a controlled vineyard (single location) for preliminary evaluation. Superior clones identified through this initial assessment are then evaluated phenotypically across multiple locations and over multiple years to ensure stability and performance. Ultimately, the best-performing clones are registered and released to the grapevine industry. Activities during Germplasm Selection are primarily conducted by grapevine breeding programmes, whereas phenotypic evaluation and final clone registration involve collaboration between grapevine breeders and national varietal offices

Nevertheless, clonal selection faces several substantial challenges, the most significant being the extensive time required for thorough performance evaluation. This time investment, typically spanning 10–15 years, is necessary to confirm the stability of desired phenotypic traits across multiple growing seasons and diverse environmental conditions; for rootstocks, the evaluation process is even longer and more labour-intensive. Another challenge arises from the perennial nature of the crop, which increases the costs associated with maintenance and phenotypic assessments. Lastly, clonal selection relies exclusively on existing genetic variability, whether naturally occurring or artificially induced, much of which has been diminished following the widespread adoption of a limited number of “elite’ clones by winegrowers. Moreover, before the evaluation phase can even begin, a considerable amount of preparatory work is required: candidate clones must first be identified, carefully selected, and assembled into a test population. This process involves labour-intensive steps such as preparing cuttings, grafting, and conducting virus tests, underscoring the monumental efforts needed prior to the actual selection program *(*Fig. 2*)*.

Studies on somatic mutations have confirmed their effect on various mostly simple traits. Berry colour represents one of the best-studied examples, with cases like a retrotransposon insertion causing the white berry phenotype (Kobayashi et al. 2004), or large chromosomal rearrangements causing the Tempranillo Blanco variant (Carbonell-Bejerano et al. 2017). Another example are two SNPs in the coding sequence of a *HAL2*-like gene that have been proposed as a likely cause for seedlessness in Corinto bianco, a somatic variant of Pedro Ximenes (Royo et al. 2015). More definitively, a SNP in the *VviAGL11* gene has been shown to cause seedlessness in the Sultanina cultivar (Royo et al. 2018). Still the genetic base of many phenotypic traits, particularly highly complex traits, remains largely unknown. However, clonal grapevine populations exhibiting substantial trait variation represent a powerful resource for studying trait genetic architectures. In some ways, and with important distinctions, clones of the same grapevine cultivar can be studied with some conceptual similarities to near isogenic lines (NILs) in other crops, while also exhibiting other features characteristic of mutant line collections. While NILs are generated through recurrent backcrossing in order to induce defined allelic introgressions, clonal variants arise from spontaneous somatic mutations that can be multiple and of diverse types, and conservation of those using vegetative propagation. Depending on the clonal lineage composition, some clones may carry unique somatic variants similar to mutant lines, whereas others share common somatic mutations inherited from the same ancestral clonal line, resulting in groups of clones with highly similar genetic backgrounds. Because most of their genetic background remains identical to the original cultivar, grapevine clones can still be highly useful for studying the phenotypic effect of these relatively few accumulated genomic alterations against a common genetic background, especially when comparing closely related clones with distinct phenotypes.

Epigenetic modifications are emerging as additional drivers of clonal differentiation, particularly in relation to phenotypic plasticity and environmental adaptation (Douhovnikoff and Dodd 2015). While research on epigenetic contributions to grapevine clonal diversity remains nascent, initial studies employing methylations sensitive molecular markers have shown it was possible to clearly separate Pinot clones based on methylation profiles; additionally studies on Malbec have found that methylation changes related to environmental stimulus are clone dependant (Ocaña et al. 2013; Varela et al. 2021). Clonal populations in grapevine thus offer an ideal framework to examine epigenetic mechanisms in perennial crops, their influence on plant phenotypes, their contribution to clonal differentiation, and their response to environmental stimuli (Berger et al. 2023).

The grapevine apical meristem, like many dicotyledonous plants, is typically composed of two different cell layers (L1 and L2) that typically remain separated. Therefore, new somatic mutations appear in only one cell layer, leading to periclinal chimeras (Pelsy et al. 2015). All periclinal chimeras are stable trough vegetative propagation, and so each cell layer accumulates molecular variants independently. This separation allows each layer to accumulate and fix its own somatic mutations, resulting in a mosaic of genetically distinct cell populations within a single plant. This genetic mosaicism, where different cell layers contributing to the plant's structure and function can possess distinct genotypes, introduces significant complexity to understanding gene expression, phenotypic outcomes, and the overall genetic basis of intra- and intervarietal variation. For instance, the final phenotype of an organ can be a composite result of interactions or differing contributions from these genetically distinct layers. With the employment of modern breeding technologies, it will be possible to overcome some of the longstanding challenges of clonal selection. For example, genomic prediction could furthermore allow to predict performance early in a plant’s lifecycle cutting the time required for evaluation (Daetwyler et al. 2013), while genome editing technologies, such as CRISPR/Cas9, offer the ability to introduce precise genetic modifications, thereby directly incorporating beneficial traits rather than waiting for them to arise naturally (Ma et al. 2016).

## Exploring phenotypic variability in clones

### Unravelling agronomical variability in grapevine clones

While early clonal selection programmes primarily focused on optimizing established viticultural traits such as yield potential and berry composition to ensure varietal typicity, contemporary efforts increasingly target traits crucial for adaptation to changing environmental conditions and evolving market demands. This includes a growing emphasis on identifying clones with delayed phenology, improved water use efficiency (WUE), enhanced disease resistance, and specific oenological profiles, alongside the traditional quality attributes. Evaluations of clones from different major *V. vinifera* varieties have revealed substantial differences in a number of important agronomic traits, especially those affecting yield potential and vegetative growth. Studies on eleven and twelve Pinot noir clones (Mercado-Martin et al. 2006; Anderson et al. 2008) showed that they differ significantly for cluster number, berry set, and total vine productivity, with yield varying 1.5-fold due to differences in berries per cluster along with variation in shoot vigour. Similarly studies on Cabernet Sauvignon (Wolpert et al. 1995), Merlot (Benz et al. 2006), and Chardonnay (Fidelibus et al. 2006) clones demonstrated huge variation in total yield through variations in cluster architecture, the number of berries per cluster, and plant vigour.

Climate change, particularly the increase in average temperatures, has been accelerating phenological development in grapevines. This shift leads to several agronomic challenges, such as an increased risk of late frost damage in some regions, earlier harvest dates, and potential declines in fruit and wine quality (van Leeuwen et al. 2024). For example, in Alsace, Riesling is now harvested approximately 25 days earlier than in 1980 (Duchêne and Schneider 2005). Given these challenges, the ability to delay phenology is an important trait for climate resilience, as it allows grapevines to shift critical developmental stages in time, mitigating the adverse effects of rising temperatures. Viticulturists currently try to delay phenological development through agronomic practices such as late pruning, oil application at bud swelling, and canopy trimming. Although effective, these methods are labour-intensive; thus, adopting clones with naturally delayed phenology offers a more resource-efficient solution. Significant clonal differences in the timing of crucial phenological events, such as bud burst, flowering and ripening, have been observed in many major cultivars. In Sauvignon blanc and Chenin blanc, variation in heat unit requirements among 263 and 187 clones resulted in different budburst and ripening times, suggesting a strong clone effect regarding phenological responses (Garcia de Cortazar-Atauri et al. 2017; Neethling et al. 2023). Similarly, an analysis of 21 Malbec clones revealed significant intra-varietal variation in flowering-to-veraison duration, with a spread of more than 16 days between early and late clones (van Houten et al. 2020). In Tempranillo studies of 30 clones revealed as much as 28 days of difference in the total vegetative cycle; interestingly, the most delayed clones showed longer veraison-to-harvest periods, which are highly desirable for climate adaptation in traditional wine regions with warmer climates (Portu et al. 2024). Such results evidence that the selection of late-ripening clones within cultivars could be an effective approach to maintaining quality in a warming climate that on average facilitates phenological development in most important grapevine cultivars.

Water use efficiency (WUE) is another important attribute for adaptation in view of the rising incidence of drought stress in viticulture. Tempranillo, Grenache, and Monastrell have been reported to exhibit substantial clonal variation in WUE at both physiological and agronomic levels. In Tempranillo, significant differences in WUE were observed across multiple years, with variability among Tempranillo clones reaching up to 80% of that observed in a grapevine cultivar collection (Tortosa et al. 2016; 2019a, b). Similar trends were reported in Grenache, where genetic differences among clones led to varying carbon assimilation strategies and stomatal behaviours (Buesa et al. 2021). In Monastrell, clonal responses to drought stress differed substantially, with certain clones demonstrating a greater ability to sustain yield and grape composition under limited water availability (Romero et al. 2023). The potential for clonal selection to enhance WUE is further supported by isotopic discrimination studies (δ^13^C), which provide a stable indicator of long-term water use patterns from veraison to harvest in grapevines (Gaudillère et al. 2002; Tortosa et al. 2019b). Given the increasing constraints on water resources, identifying and propagating high-WUE clones in combination with the use of drought-resistant rootstocks represents a promising avenue for sustaining viticulture in drought-prone regions.

Another crucial aspect of climate adaptation is the selection and adoption of superior clones with improved disease resistance. As climates warm, some historically significant wine regions will likely experience an increased incidence of fungal diseases. This challenge is particularly relevant in Europe, where the European Green Deal aims to reduce chemical pesticide use by 50% by 2030 (European Commission 2019). Due to its high susceptibility to fungal diseases, grapevine cultivation relies heavily on fungicides, with vineyards typically requiring 10 to 15 fungal treatments per year. The exploitation of genetic diversity in susceptibility is one potentially interesting aspect of more sustainable vineyard management. It has been reported that 10 different clones of Cabernet franc showed different susceptibility levels to downy mildew (*Plasmopara viticola*); the lower susceptibility levels were linked to a higher production of stilbenic phytoalexins involved in downy mildew resistance mechanisms (van Leeuwen et al. 2013). In a study analysing eight clones of Albariño, differences in the size and number of stomata have been connected to variability in susceptibility for downy mildew (Alonso-Villaverde et al. 2011). Clonal variation significantly influences susceptibility to Botrytis bunch rot in grapevines. In particular, clones with looser clusters and smaller berries exhibit lower susceptibility due to improved airflow and reduced berry-to-berry contact, both of which limit infection spread. Such clonal variants with distinct cluster architectures have been documented in numerous grape varieties, including Albariño, Riesling, Pinot gris, and Tempranillo. Across these varieties, clones characterized by looser clusters consistently show reduced Botrytis incidence (Alonso-Villaverde et al. 2008; Molitor et al. 2018; Belfiore et al. 2024; Portu et al. 2024). In Pinot gris specifically, it has been demonstrated that a combination of looser cluster architecture and thicker berry skin can decrease Botrytis infection rates (Belfiore et al. 2024). These findings align with transcriptomic studies that have identified key gene networks related to cell wall composition and hormone signalling, which regulate cluster architecture and disease susceptibility (Grimplet et al. 2017, 2019).

The results of these studies emphasize the potential of clonal selection as a tool for climate adaptation. Yet opportunity exists for future research to compare the extent of trait variation between varietal clonal populations in order to determine if there is greater selection potential in one variety versus another for target traits. This is particularly relevant given that clonal selection can either stabilize or diversify phenotypic distributions depending on historical selection pressures, trait heritability, and the role of somatic mutations in maintaining variation. Understanding these dynamics across varietal clonal populations may help identify varieties where within-clone variation is most exploitable for genetic improvement. That said, the phenotypic diversity reported to date in grapevine clonal populations represents an invaluable resource for adaptation to environmental challenges while preserving regional typicity and wine quality.

### From vine to wine: how clonal variation shapes wine characteristics

Considerable attention has been given to the impact of clonal variation on agronomic performance in the vineyard; however, equal emphasis should be placed on its influence on the final product, the wine. Genetic contrasts between clones lead to changes in bio-chemical pathways that create different metabolic profiles, impacting concentration of sugars, acids, phenols, and aromatic compounds, which all contribute to the final wine quality. Yet studies about clone effects on wine traits remain sparse compared to research on agronomic performance. The main reason is the complexity of characterizing wine traits which usually involves expensive and laborious processes including micro-vinification, sensory evaluations, or costly analytical techniques for metabolite profiling.

Research in different cultivars has shown major clonal differences in chemical makeup, especially in phenolic and volatile profiles. A comprehensive analysis of 27 clones from eight *V. vinifera* varieties demonstrated significant intra-varietal variability in both grape and wine phenolic content (Ren et al. 2023). Another study of 131 Malbec clones found significant variations in anthocyanin profiles, mainly due to variations in concentrations of malvidin derivatives. These changes have been shown to be connected with different expressions of genes *F3′5'H*, *OMT1* and *AM2*, all gene involved at different steps of anthocyanin biosynthesis pathway (Muñoz et al. 2014). Similarly, different Tempranillo clones showed 2.4-fold differences in anthocyanin content (Royo et al. 2021). These studies demonstrate that variations between clones can be just as important as differences between grape varieties in how they affect phenolic composition.

The analysis of volatile compounds reveals differences among clones as shown in Sauvignon blanc plus Cabernet Sauvignon varieties. A really noticeable pattern emerged in Sauvignon blanc where clone SB316 contained more fruity thiols and esters. But clone SB11 showed higher methoxypyrazine amounts which led to greener and herbaceous scents (Šuklje et al. 2016). The research on Cabernet Sauvignon clones 169 and 191 displayed varied volatile compound levels. Clone 169 created wines with richer floral scents along with roasted flavours during oak ageing. Clone 191 exhibited more chemical as well as green aroma traits (Qian et al. 2022). Chardonnay clone 809 is widely recognized as a unique variant among Chardonnay clones, known for its aromatic profile resembling muscat grapes. This results in Chardonnay wines with enhanced floral and sweet notes. Research has shown that this distinct aroma is due to a higher abundance of monoterpenoid compounds compared to non-aromatic clones. (Bernardo et al. 2022). Notably, this specific aroma profile and increased monoterpenoid content have been linked to a missense mutation (S272P) in the *VvDXS1* gene, which was identified in Chardonnay clone 809 (Roach et al. 2018).

The evolution of phenolic compounds and antioxidant activity during bottle ageing has also been shown to differ among clones. In Cabernet Sauvignon, wines produced from clones 169 and 685 had different total phenolic content and different chemical composition connected with colour characteristics. These clonal differences persisted during 11 months of bottle ageing. These findings underscore how clone-specific phenolic compositions can shape both sensory attributes and wine stability over time (Burin et al. 2011). In White Riesling, clones 198 Gm and 239 Gm exhibit higher concentrations of terpenes such as nerol, contributing to rose and citrus aromas. Clones 24 Gm, 94 Gm, and 110 Gm are rather neutral in aroma, while clone 64 Gm shows elevated levels of linalool, geraniol, and trans-2-hexenal, resulting in aromatic wines (Schmid et al. 2019).

Also, the concentration of secondary metabolites, such as flavanols and hydroxycinnamic acids, has been identified as a differentiator among clones. One study on Muscat of Alexandria biotypes revealed that Spanish biotypes presented higher levels of flavanols and hydroxycinnamic acids, compared to other biotypes, contributing to unique aromatic profiles (De Lorenzis et al. 2017). In four certified Duras N clones, variation was detected in rotundone concentrations, a compound responsible for peppery aromas in wine, suggesting that clones with greater disease resistance may synthesize higher levels of specific aroma compounds in response to biotic stresses (Geffroy et al. 2015).

## Unravelling the molecular basis of clonal variation

### Early insights into clonal diversity from molecular marker studies

Molecular markers have been initially applied in grapevine fingerprinting since the 1990s, with the use of random amplified polymorphic DNA (RAPD) (Jean-Jaques et al. 1993), amplified fragment length polymorphism (AFLP) (Sensi et al. 1996), and simple sequence repeat (SSR) (Thomas and Scott 1993).

These marker technologies were also trialled for detection of somatic mutations and estimation of intra-varietal variability. One of the first attempts, by Cipriani et al. (1994), employed a set of 5 different SSR to differentiate between 16 cultivars and 42 clones, while it was possible to correctly identify all the examined cultivars the microsatellite repeats failed to distinguish clones in all cultivar. On the other hand, research that employed 8 AFLP and 5 inverse sequence-tagged repeat (ISTR) markers on 13 clones of Sangiovese detected polymorphisms within the cultivar; this demonstrated the potential of molecular markers as a tool in the investigation of genetic diversity within varieties (Sensi et al. 1996).

As molecular marker technologies advanced, improving their resolution, clonal identification became more precise through the increased number of available markers and the integration of different marker approaches. For example, it was possible to disclose genetic differences mainly in chimeric state, in a collection of 145 pinot clones with a set of 49 SSR markers (Hocquigny et al. 2004). In another study, RAPD, SSR and I-SRR were used in differentiation and identification of 10 clones of white Riesling: whereas the RAPD markers were found not stable for the purposes of identification, the markers SSR and Inter-SSR yielded reproducible genetic profiles which allowed clone differentiation with high confidence (Regner et al. 2000). In contrast, a study using SSR, AFLP, and methylation-sensitive amplified polymorphism (MSAP) markers in 24 clones of the cultivar 'Traminer' found that clonal differences by SSR markers could not be detected using SSR markers alone. However, AFLP markers allowed the identification of 16 out of the 24 clones, MSAP markers, on the other hand detected epigenetic variations, and proved that epigenetics could play a major role in clone differentiation as well (Imazio et al. 2002).

Building on these, a multivariate approach was then developed for investigating intra-varietal genetic diversity and clonal distinction in *Vitis vinifera* with AFLP, SAMPL, M-AFLP, and I-SSR markers. This allowed for the establishment of distinct clones within several cultivars but also permitted the search for correlations between molecular profiles and geographic origins, where genetic divergence between accessions from different regions had occurred despite their historical classification as a single variety (Meneghetti et al. 2012).

Despite these advances, molecular marker-based clonal identification still has its limitations. Genetic differences between clones are often subtle, since they result from rare somatic mutations are often not detectable. Further complicating this issue are chimerism and epigenetic modifications that molecular markers alone may not fully capture.

### Decoding clonal variation with whole-genome sequencing

Initial assemblies of model plants were essential in laying the foundation to understand genome organization and evolution, which again led the path for similar efforts in economically important crops. The publication of the first reference genome for *Vitis vinifera* (Jaillon et al. 2007) marked a milestone in grapevine genomics, enabling the identification of genetic factors determining important agronomical traits and to enlighten clonal differentiation mechanisms. With these new tools, it started to be possible to answer questions such as: What’s the extent and the nature of genetic variability within cultivars? Which part of the genome is the most affected by somatic variants? Is it possible to reconstruct clonal lineages demonstrating how selection for important traits could have shaped clonal diversification?

Once the reference genome had been established, molecular tools started to develop quickly, such as the 18 k SNP chip (Le Paslier et al. 2013). De Lorenzis et al. (2017) used genotypic data obtained with this chip and attempted to discriminate between 15 clones of Aglianico and 21 clones of Muscat of Alexandria. Only few minor genetic differences among clones were detected, indicating that genome-wide methods are essential to capture subtle somatic mutations effectively.

Subsequent whole-genome analyses revealed extensive genetic variability within grape cultivars. Carrier et al. (2012) compared three pinot clones approximately over a genomic region of 4.5 Mb and demonstrated the significant role of transposable elements, particularly Gypsy-like elements, by identifying 147 polymorphic insertion sites across just 1% of the genome. Extensive genomic analysis using Illumina whole-genome sequencing comparisons has been executed by Gambino et al. (2017), who analysed three certified Nebbiolo clones and identified 28,870 SNPs among them, Roach et al. (2018), who found 1620 high-confidence marker variants across 15 Chardonnay clones, and Vondras et al. (2019), who reported 507,495 SNPs and 100,424 InDels, of which 13% and 16% were private to one or two clones, and 6,340 transposable element insertions (TEI) among 15 Zinfandel clones. These studies demonstrated that most somatic mutations occur in non-coding regions, particularly in repetitive and intronic areas, and highlighted the notable contribution of TEIs in regulatory regions with potential effects on gene expression and clonal diversity.

Clonal lineage reconstruction has further illustrated how selection pressure shapes genetic diversity in clones. Gambino et al. (2017) successfully classified Nebbiolo clones into geographically structured groups based on SNP markers, while Calderon et al. (2021) linked Malbec lineages to distinct clonal propagation histories in Argentina and Europe. Roach et al. (2018) showed that phenotypically similar Chardonnay clones clustered together, supporting bud sport derivation.

These studies together confirm that next-generation sequencing has completely changed the way investigation into clonal variation is conducted in grapevines. One result that seems to emerge from several investigations is that somatic SNPs and small InDels are mostly heterozygous, mostly in intergenic regions, and generally of low predicted impact. However, there is great variation in the number of reported variants, and this difference can be accounted for by biological factors, such as the number of clones analysed and the age of the cultivar, as well as by technical factors including bioinformatic pipelines used, filtering stringency, and choice of reference genome.

Most studies have given less emphasis to structural variants of a larger size (> 50 bp) due to the inherent limitation of short-read sequencing technologies; recent genomic advances such as the development grapevine pangenome and subsequential analysis highlighted how structural variants significantly contribute in shaping economically important traits (Liu et al. 2024). Additionally, the widespread use of a haploid reference genome in the analysis of a highly heterozygous species like grapevine has led to an overestimation of heterozygous loci that may not correspond to genuine clonal differences, while potentially overlooking true somatic variant (Wang et al. 2024). In addition, these studies have provided limited insights into the potential contribution of epigenetic modifications in clonal diversification.

Recent advances in the long-read sequencing platforms, such as PacBio and Oxford Nanopore Technologies (ONT), show potential to overcome many of these issues. These systems give longer read lengths that could permit more complete characterization of structural variation and direct phasing of heterozygous regions. This increased resolution not only makes it possible to better evaluate somatic variation but also opens up the possibility of integrating epigenetic data, thus providing further insights into mechanisms driving clonal diversification in grapevines across multiple biological layers.

### How somatic variants shape grapevine phenotypes

Berry colour is the most extensively reported and exploited phenotypic change in grapevine clones. It is now knowledge that berry colour is regulated by a cluster of four *MYB*-type transcription factor genes, *VvMybA1-VvMybA4* present on a ~ 200-kb genomic region on Chromosome 2 (Azuma et al. 2009). Mutant clones of red-fruited varieties that have lost function in these genes and exhibit grey or white berries are recorded for many varieties. Pinot has various somatic variants exhibiting all range of different berry colours; these changes are caused by different insertion and deletion events or a retrotransposon insertion, denominated *Gret1*, insertion affecting the transcription of *VvMybA1* (Pelsy et al. 2015). Similarly, in the red cultivar Cabernet Sauvignon a hemizygous deletion of at least 260 kb removing the whole region, originated white and bronze-berried variants (Walker et al. 2006). Interestingly, in Tempranillo the colour switch is caused by a massive hemizygous deletion affecting 313 genes (Carbonell-Bejerano et al. 2017). While the switch from red berries to white/grey berries is represented by a gene loss function, also the opposite transformation, from white-berried mother plants to red or grey berried mutants is recorded, showing how somatic variants could actually originate gain of function. It was shown in Muscat of Alexandria and Italia how red-berried variants are originated from the recovery of expression of *VvmybA1*, thanks to a intra-long terminal repeat (LTR) recombination within *Gret1* (Kobayashi et al. 2004). In Benitaka, *Gret1* is still present, but the expression of *VvmybA1* is restored thanks to an homologous recombination event between *VvmybA1* and *VvmybA3*, and in particular by the introduction of a promoter sequence deriving from *VvmybA3* which contains two ABRE-like sequences (Azuma et al. 2009). In Riesling instead, a 67-kb deletion, containing *Gret1*, merged together *VvmybA1a* and *VvmybA3* originating a functional version of *VvmybA1* (Röckel et al. 2022).

Somatic mutations also shape grape cluster architecture, an important trait connected to Botrytis infection susceptibility. In Pinot noir, variations in bunch compactness, in particular in pedicle length, have been traced to single-nucleotide somatic mutations in the *VvGRF4* gene, a growth-regulating factor targeted by microRNA *miR396*. Alterations in the *miR396* binding site stabilize *VvGRF4* transcripts, leading to enhanced rachis and pedicel elongation (Rossmann et al. 2020).

Deleterious phenotypes can also emerge from somatic variants, the fleshless berry (*Flb*) mutation in Ugni Blanc, is caused by a transposon insertion in the promoter of *VvPI* (a PISTILLATA-like MADS-box gene), results in ectopic expression of floral identity genes during early fruit development. This misexpression disrupts normal cellular differentiation, leading to a clone with the formation of seed-containing but flesh-deficient berries (Fernandez et al. 2013).

### Exploring the role of epigenetics in clonal diversification

While genetic variation underpins the basis of plant diversity, epigenetic modifications introduce another layer of variation that is dynamic. Epigenetics broadly encompasses mitotically, and sometimes meiotically, heritable changes in gene activity, and sometimes phenotype, that occur without direct alterations to the underlying DNA sequence (Berger et al. 2009). Epigenetic modifications include DNA methylation, histone modifications, and small RNA-mediated gene regulation, all contributing to the regulation of plant development, stress response, and phenotypic plasticity (Pikaard and Mittelsten Scheid 2014).

A particularly fascinating feature of epigenetic regulation is its remarkable sensitivity to changes in the environment. While the emergence of new genetic mutations is a largely stochastic process, with rates and locations influenced by factors such as DNA sequence context, chromatin state, and repair efficiencies (Monroe et al. 2022), the subsequent selection and stabilization of somatic variants leading to phenotypically distinct and stable clones are a gradual process that can unfold over decades or even centuries of vegetative propagation. In contrast, epigenetic marks can be induced or erased much more rapidly, often in direct response to external conditions such as temperature fluctuation, drought stress, or field management (Fortes and Gallusci 2017).

It is suggested that epigenetic regulations play a huge role in the adaptation of perennial species and clonal crops, with limited genetic variation, to changing environments (Sáez-Laguna et al. 2014; Sammarco et al. 2022). Additionally, recent studies indicate that the performance of a clonally derived individual is shaped not only by its current conditions but also those experienced by its “parent”, and this transgenerational effect can be attributed to some stable epialleles (Huber et al. 2021; van Antro et al. 2023). The persistence of specific epigenetic markers in clonal lines has been observed in fruit crops such as apple, where bud mutants are often linked to methylation changes rather than genetic mutations (Du et al. 2020).

In a study on three Malbec clones grown in two vineyards with contrasting environments, researchers found phenotypic variation among clones and sites despite low genetic variation. After analysing genomic regions with methylation-sensitive amplification polymorphism (MSAP), they attributed these to phenotypic plasticity and epigenetic regulation. What is more, their results showed that the most intense factor affecting methylation pattern changes was the genotype; therefore, these epigenetic modifications might be clone-dependent (Varela et al. 2021). To further investigate, the researchers collected cuttings from one of the clones in both vineyards and cultivated them in a common vineyard, analysing methylation patterns over three growing seasons. During the initial season, differences in methylation were still detectable, likely reflecting the memory of the previous environments. However, these differences gradually diminished over time, suggesting a reprogramming of the hemimethylated patterns following transplantation into the common vineyard (Varela et al. 2024).

The first evidence of epigenetic variation among grapevine clones was obtained using MSAP. Already in the early 2000s, it was possible to prove that epigenetic changes might distinguish clones from the same cultivar (Imazio et al. 2002). Moreover, MSAP analysis of Pinot noir clones identified stable epigenetic polymorphisms, which were capable of distinguishing 92.5% of the clones analysed, reinforcing the idea that epigenetic variation might be a key player in clonal diversification (Ocaña et al. 2013).

Recent studies have shown that epigenetic modifications may drive phenotypic variation in clones even without genetic changes. In Brazil, a bud sport of Benitaka, the demethylation of the 3′ LTR region of a retrotransposon (*Gret1*) in the promoter region of the *VvMYBA1* gene was linked to a striking increase in anthocyanin accumulation, darker berry skin, and red-fleshed fruit (Azuma and Kobayashi 2022).

Somaclones, or plants derived from somatic embryogenesis, exhibit epigenetic variability, often arising due to in vitro culture conditions. These variations, particularly in DNA methylation patterns, have been observed in multiple plant species, including grapevine, where they contribute to intra-clonal diversity (Carra et al. 2024). In a study, 78 somaclones derived from somatic embryos of two distinct cultivars were analysed using AFLP and MSAP. MSAP revealed DNA methylation differences among somaclones and their respective mother clones, demonstrating how in vitro culture via somatic embryogenesis can introduce new variations into old cultivars without changing their main characteristics (Schellenbaum et al. 2008). Using genome-wide bisulphite sequencing and RNA sequencing, researchers observed a significant increase in genome-wide CHH methylation in embryogenic callus, particularly within heterochromatic regions. This de novo methylation correlates with increased transcript abundance from highly amplified transposable element (TE) families and associated 24-nt heterochromatic siRNAs. These findings provide insights into the genomic mechanisms underlying somaclonal variation and epiallele formation in plants regenerated from embryogenic cultures (Lizamore et al. 2021).

These findings highlight the critical role of epigenetic mechanisms in clonal diversification, adding an extra layer of variation beyond genetic differences. The stability of some epialleles across clonal generations suggests that epigenetic regulation could be used to refine clonal selection strategies with the aim of improving traits related to stress resilience and adaptation in perennial species (Berger et al. 2023). With evidence from available studies, it is obvious that epigenetics plays an important role in shaping clonal variation, but the understanding of its mechanism is far from being complete. Clearly, much additional work will be required to untangle complex interactions between genetic background, environment, and epigenetic modifications.

### Cell layer-specific molecular variants as a driver for intra- and inter-clonal diversity

In higher plants, the shoot apical meristem comprises three distinct layers with specialized functions. The outer L1 layer is made up of epidermal cells produced by anticlinal divisions, beneath it, the multicellular L2 layer generates all subepidermal tissues through multidirectional divisions that yield both primary structures and secondary meristems, in some species, an inner L3 layer forms the pith, although this layer has not been definitively identified in grapevine (Thompson and Olmo 1963; Torregrosa et al. 2019). Recent research in apricot trees indicates that somatic mutations in plants are highly layer-specific, with over 90% of mutations confined to individual meristematic layers. Interestingly, the L1 layer exhibits a higher mutational load compared to L2, suggesting distinct mutational dynamics between layers, likely influenced by differences in genome integrity control mechanisms and potentially compounded by the L1 layer's greater exposure to environmental mutagens, such as UV radiation (Goel et al. 2024).

When somatic modifications occur in a single meristem cell belonging to a specific cell layer, the mutant cell may proliferate and colonize that layer, resulting in a mericlinal chimera. If this colonization becomes complete, such that every cell in the layer carries a mutation absent in the other layers, a periclinal chimera is formed. Periclinal chimeras are the most stable and can be propagated via clonal methods (Frank and Chitwood 2016). During genetic profiling of grapevine cultivars, certain individuals have exhibited more than the expected two alleles at specific loci. Detailed analysis has revealed that this is attributable to periclinal chimerism, where distinct cell layers in the apical meristem contribute divergent genetic profiles (Franks et al. 2002).

Pinot meunier, a commercially important cultivar in Champagne production, serves as the most thoroughly studied example of periclinal chimerism in grapevine. As a somatic variant of Pinot noir, it displays distinctive tomentose (densely trichome-covered) shoot tips and emerging leaves. This phenotype has been linked to a SNP in the DELLA domain of the *VvGAI1* gene (Boss and Thomas 2002). To substantiate its chimeric structure, researchers separated the two apical meristem cell layers via somatic embryogenesis. The regenerated plants exhibited divergent DNA profiles and new morphological characteristics: Those originating from the L1 layer demonstrated dwarfism along with the hairy leaf phenotype typical of Pinot meunier, whereas plants derived from the L2 layer were indistinguishable from Pinot noir (Franks et al. 2002).

A recent genomic study by Sichel et al. (2023) not only expanded our understanding of cell layer differentiation but also demonstrated that genetic variations between cell layers in grapevines can currently be detected via long-read sequencing. By using DNA from leaves (L1 + L2) and DNA from roots (L2-only), the researchers obtained different genome assemblies of Merlot; 51 and 53 layer-specific variants were mapped across the genome in the two haplotypes, some directly affecting the coding regions of key genes involved in development and stress response.

Chimeric structures are generally thought to be extremely stable, evidenced by the continuous successful propagation of Pinot meunier in France since the seventeenth century. However, instability has been observed, as reversion events from Pinot meunier to Pinot noir are reported quite frequently (Franks et al. 2002). The basis for such phenotypic instability is thought to be cellular rearrangements within the chimeric state. This may include a process called "displacement", in which cells from the inner layers invade the outer L1 layer due to the inner layer's less controlled pattern of cell division.

While chimerism complicates clonal selection in grapevine, it is also a very interesting biological phenomenon with implications for viticulture, evolutionary biology, and functional genomics. Furthermore, the interaction between genetic mosaicism and phenotypic expression is crucial for optimizing grapevine breeding and selection strategies. It is also reasonable to expect that cell layer-specific differences may extend beyond genetic variants to include epigenetic differences, further influencing phenotypic outcomes. Future research is needed to better understand the interplay between layer-specific genetic and epigenetic variants and their effects on agronomically important traits.

## Breeding the future: strategies to leverage clonal diversity

### Limitations of clonal selection

Despite the great results of clonal selection in commercializing new grapevine clones with more favourable trait characteristics, such as decreased disease susceptibility, delayed phenology, and quality parameters, conventional clonal selection is notoriously slow and inefficient. Besides the fact that the identification and evaluation of superior clones often require very long periods, often exceeding decades in order to determine the stability of specific characteristics across a range of different environments (Torregrosa et al. 2011), several key limitations to the process exist that can inhibit both short- and long-term genetic gains. Clonal selection depends exclusively on existing genetic variability, which can be naturally or artificially induced. This makes the process highly dependent on chance that (i) new genetic variation with useful effects on viticultural traits arises and that (ii) these new useful variants that can be highly tissue- and/or even cell layer-specific can be reliably identified and propagated. Even if successfully identified, putatively useful new chimeras may be phenotypically unstable; for instance, the reversion from Brazil to Benitaka was estimated at 28.57% (Oliveira Collet et al. 2005). Within the framework of clonal selection, recombining alleles across genetic backgrounds via targeted hybridization is not feasible and, consequently, the possibilities of creating novel desired trait configurations are highly limited. For newly developed variety clones to gain official approval from the relevant varietal authorities, they must adhere to predefined phenotypic thresholds. Deviations beyond these limits may result in reclassification as a distinct variety (International Organisation of Vine and Wine 2017).

Modern breeding tools and technologies, like predictive breeding and genome editing, provide new avenues to substantially accelerate the process of conventional clonal selection.

### Improving clonal selection with predictive breeding

Recent advances in genomic selection and predictive breeding provide a promising pathway to overcome the inefficiencies of traditional clonal selection. In broader grapevine breeding contexts, such as in diversity panels or in structured populations originating from targeted crosses, leveraging high-throughput sequencing and large-scale phenotypic datasets has shown promise for developing models to predict performance, reducing reliance on lengthy field evaluations(Brault et al. 2024). The direct translation of current predictive approaches to clonal selection within established cultivars is not straightforward and, to our knowledge, has not yet been successfully implemented. The nature of clonal variation itself presents primary challenges. For instance, clones of the same cultivar are typically genetically very similar. This might result in low overall genetic diversity, with differences usually arising from a relatively small number of somatic mutations. Many of these impactful somatic mutations are also mainly rare and often private to a specific clone or a narrow clonal lineage. This is unlike more diverse crossbred populations, where many allelic variants are commonly shared. As a result, conventional models, which depend on widespread linkage disequilibrium across diverse training populations, might be not well suited for clonal populations.

Nevertheless, potential may exist for specific scenarios, particularly with older cultivars that have accumulated greater intra-varietal diversity over extended periods of propagation that underpin trait variation and is shared across multiple clonal lineages. If large, well-structured reference populations of such clones can be assembled, encompassing a broader spectrum of existing somatic variants and detailed phenotypic data, it might become feasible to develop tailored predictive models. Furthermore, the use of whole-genome data can significantly enhance the accuracy of genetic merit estimates for newly identified variants, ensuring that resources are allocated to germplasm with a high likelihood of superior performance. Additionally, advancements in sequencing technologies now allow for precise differentiation between clones and even among cell layers within a clone, facilitating the translation of predictive breeding approaches from crossbreeding programmes into clonal selection strategies. The principle of genomic selection can also be extended to include not only genetic but also epigenetic markers, e.g. as demonstrated in a recent study in dairy cattle (González-Recio et al. 2023). This seems particularly interesting when novel sequencing technologies like ONT are used that can simultaneously generate genetic and epigenetic data. Predictive breeding to accelerate clonal selection is likely particularly effective for complex traits influenced by multiple genes, whereas its applicability may be more limited for monogenic traits where single mutations can have a significant impact. This limitation is especially relevant for novel mutations, as their effects cannot be predicted due to their absence in existing reference datasets. The potential utility of emerging predictive breeding approaches, such as phenomic selection (Brault et al. 2022), remains to be determined. Their effectiveness will largely depend on the resolution of the phenomic data acquisition platform and its ability to distinguish between clones of the same cultivar.

### Leveraging clonal populations to explore grapevine trait genetics

Near isogenic lines (NILs) are specialized genetic stocks in which nearly the entire genome is identical except small chromosomal regions. By repeatedly backcrossing a donor, usually a wild relative with an elite adapted cultivar, the recurrent parent, and using molecular markers to select for the introgressed region, researchers can generate NILs in which any observed phenotypic differences are attributable solely to that specific genomic segment (Tuinstra et al. 1997). NILs provide a powerful platform for fine mapping and positional cloning because the confounding background noise is minimized, thereby enabling a precise dissection of complex traits via direct correlations between genotype and phenotype for important traits (Monforte and Tanksley 2000). NILs have been successfully used to discover QTLs in a wide range of crops, including tomato, maize, and wheat (Eshed and Zamir 1995; Szalma et al. 2007; Mia et al. 2019).

In perennial fruit trees, the development of NILs has been extremely limited. This is largely attributable to two major challenges: first, the process is inherently time‐consuming and costly given the extended generation times of these species, and second, selfing or backcrossing tends to induce severe inbreeding depression in these highly heterozygous plants, making it impractical to obtain NILs. In this context, clonal populations of the same cultivar offer a valuable, but still distinct, resource for studying the genetic basis of trait variation, conceptually paralleling some utilities of NILs while differing fundamentally in origin and the nature of their genetic variation. While NILs are intentionally created to isolate one or a few defined introgressed genomic regions against a recurrent parent background, grapevine clonal populations arise from the accumulation of spontaneous somatic mutations over long periods of vegetative propagation. These mutations can be of various types (e.g. SNPs, InDels, structural variants, transposable element insertions), potentially multiple in number, and initially uncharacterized in their specific genomic locations and overall impact. Unlike the targeted introgressions in NILs, the genetic differences between clones within a cultivar are the product of natural variation and selection during propagation. Depending on the clonal lineage composition, some clones may carry unique somatic variants similar to mutant lines, while others share common mutations inherited from the same ancestral clonal line, creating groups of clones with highly similar genetic backgrounds. Despite these differences, the largely identical genetic background shared by clones of an old cultivar provides a powerful framework for dissecting the phenotypic effects of the relatively few distinguishing somatic variants, somewhat similar to NILs facilitating the study of specific loci by minimizing background genetic noise.

In the past, genomic and molecular studies on clonal populations in grapevine have been limited in scale, likely due to technological constraints. As a result, their application in statistical genetics has remained challenging. However, recent advancements in sequencing and phenotyping technologies now allow for higher-resolution and large-scale analyses, overcoming these previous limitations. To date, no studies have systematically leveraged these innovations to investigate trait genetic architectures in grapevine clonal populations, but doing so could provide valuable new insights. Coordinated efforts across multiple cultivars would not only enhance our understanding of clonal variation and genetic architectures of commercially important traits but could also help identify promising targets for genome editing to accelerate precision breeding in grapevine.

### Designing the ultimate clone using genome editing

Modern biotechnology tools, in particular CRISPR/Cas systems, have transformed plant genetics research by enabling precise and efficient manipulation of the genome, thus accelerating the development of superior cultivars with enhanced agronomic traits (Ma et al. 2016; Hickey et al. 2019). Gene editing can serve both to functionally validate genes and to directly generate superior genotypes. In grapevine, CRISPR/Cas systems have been successfully applied for both purposes. For example, Li et al. (2020) used knockout lines to demonstrate the role of *VvPR4b* in defence mechanisms against downy mildew, and Clemens et al. (2022) showed that *VvEPFL9-1* regulates stomatal density, thereby influencing water use efficiency. More recently, base-editing techniques have also been documented in grapevine. Yang et al (2024) employed prime editing to substitute a lysine with an asparagine at position 284 in the *VvDXS1* protein, previously identified as a major factor in muscat flavour production, resulting in edited plants with higher monoterpene levels in their leaves compared to the control.

While traditional clonal selection depends on naturally occurring genetic variation, CRISPR/Cas systems allow for the targeted creation of customized clones, eliminating the barrier to allele flow among clones. This approach begins by investigating the genetic diversity within a clonal population of a particular variety to pinpoint specific alleles or mutations responsible for desirable traits. Next, among these genetically characterized clones, those already displaying favourable characteristics are selected. CRISPR/Cas systems are then applied to precisely modify the genetic loci of interest, enabling the introduction of additional beneficial traits, such as delayed phenology, improved cluster architecture, or enhanced stress resistance, into these superior clones (Giacomelli et al. 2023).

However, a significant challenge arises when applying genome editing technologies to established periclinal chimeras. Plant regeneration, a necessary step in most genome editing protocols and often reliant on somatic embryogenesis, originates from a single cell layer (Carra et al. 2024). This process usually resolves the chimera, failing to preserve the multi-layered genetic structure essential for the unique phenotype of certain clones. For instance, attempting to genome-edit Pinot Meunier, a well-characterized L1-mutated chimera of Pinot noir where the L1 layer carries a mutation in the *VvGAI1* gene (Boss and Thomas 2002), would likely result in either an edited Pinot noir (if regeneration occurs from the L2 layer) or an edited dwarf plant with the L1-genotype (due to the *VvGAI1* mutation's effect on growth when non-chimeric), rather than a Pinot Meunier plant retaining its characteristic tomentose phenotype. This illustrates a practical limitation for applying genome editing to maintain or improve specific traits in certain historically important clonal variants.

Additionally, in any effort to create these superior clones, it is critical to preserve the variety’s core identity as defined by a number of phenological, agronomic, and quality trait characteristics. Once the distinguishing characteristics defined in its official registration are altered beyond recognition, the resulting clone can no longer be registered as a clone of the original variety (Töpfer and Trapp 2022). The key bottleneck in adopting new genomics techniques is still the legislative aspect; today, within the EU, plants obtained through new genomic techniques, such as CRSPR/Cas systems, are still strictly regulated as genetically modified organisms. In July 2023, the European Commission presented a draft regulatory that regulates Category 1 NGT plants as equivalent to traditionally bred cultivars. Still, the bill must undergo a lengthy legislative procedure before it can be adopted as a law (Ren et al. 2024).

CRISPR/Cas-based technologies will be driving further advances in grapevine genome engineering and a path to superior clones. Meanwhile, much work is still to be done in surmounting the regulatory hurdles and market acceptance by transparent communication of safety, benefits, and ethical considerations. In the end, overcoming such barriers will further advance sustainable innovation in viticulture. It is important to note that the use of NGT doesn’t play a role in speeding up the process of evaluation and registration of new clones that might still take up to 15 years even with the introduction of these new technologies, but make the whole process more precise and thus resource efficient.

### Grapevine clones as a model to explore intra-varietal selection dynamics that shape complex trait variation

Micro-evolutionary processes describe the change of populations over time through genetic mutations, selection pressures, recombination, hybridization, and epigenetic modifications. However, in clonally propagated species like grapevine, where major cultivars have been under directional selection for hundreds to thousands of years, somewhat similar processes occur through different mechanisms and could be better described as intra-varietal refinement driven by human selection.

The study of grapevine clonal populations represents a unique opportunity to investigate intra-varietal selection dynamics as they occur largely independent of sexual recombination, yet underpin and shape variation for complex traits. Building on established insights into the accumulation of somatic mutations and the role of epigenetic regulation, grapevines clonal populations allow for the investigation of mutation, genetic drift rates, and the long-term outcomes of epigenetic changes under natural and artificial selective pressure in the context of complex trait variation.

Insights obtained from grapevine clones might advance our knowledge in viticulture and could also be broadly generalized to other clonally propagated crops, such as banana, potato, cassava, and various fruit trees. By way of taking into consideration how selection pressures, somatic mutations, and epigenetic changes determine genetic diversity across generations, it is possible to acquire information on the adaptive value of such processes and their role in shaping variation landscapes for traits that are important for the industry. Furthermore, this approach can be extended to the "multi-layer" phenomenon, whereby grapevine serves as a model to study non-additive interactions between genes and how new mutations within and across cell layers might interfere with existing genetic components, especially concerning complex traits.

Additionally, grapevine clones represent an exceptional model for understanding how clonally propagated perennial plants adapt to specific environmental conditions through the accumulation of genetic and epigenetic variants. By observing how particular genetic and epigenetic changes correlate with the origin of different clones, it may be possible to identify mechanisms underlying adaptation in clonally propagated species.

## From insights to impact: the future of clonal selection in grapevine

Clonal diversity in grapevine represents a fundamental resource for improving key agronomic and oenological traits within commercially important traditional grapevine cultivars, like Pinot noir, Grenache or Chardonnay. Intra-varietal variability is driven by the accumulation of somatic mutations and epigenetic modifications, and likely their interaction, affecting yield potential, phenology, stress tolerance, and wine composition. Traditional clonal selection has been instrumental in identifying superior clonal variants, but its reliance on naturally occurring mutations and lengthy evaluation periods limits its efficiency with regards to delivering genetic gain for the viticulture industry in the future.

Recent advances in both genomics and epigenetics have dramatically improved our ability to study the molecular drivers underlying clonal diversification. The growing accessibility of long-read sequencing technologies such as Oxford Nanopore and PacBio will help to shed light on the contribution of structural variants, transposable elements, and differentially methylated loci to clonal diversity and their impact on commercially important traits.

The integration of high-throughput phenotyping with genomic and epigenomic data will enable the precise characterization of trait variation and identification of causal variants. This would have great potential for predictive breeding, whereby the agronomic and oenological value of a new clone could be predicted based on its genetic and epigenetic profile. Predictive breeding methods might reduce the time for clonal evaluation drastically and overcome one of the major bottlenecks of traditional clonal selection.

Beyond that, genome-editing technologies such as CRISPR/Cas9 will offer new possibilities to further refine and optimize clonal traits at levels of precision previously unimaginable. In contrast with classical selection that relies on spontaneous mutations, genome editing allows controlled introduction of target modifications in order to fine-tune characteristics, e.g. for fruit composition, without altering the cultivar type.

Clonal diversity in grapevine represents offers a fascinating biological system to explore how somatic mutations, epigenetic modifications, and selection pressures interact to shape complex traits variation over time. In that sense, grapevine can serve as a model for understanding intra-varietal refinement driven by human selection in clonally propagated perennials, with many research questions yet to be addressed. As modern technologies continue to evolve, there is huge potential to drive both applied innovation in breeding and fundamental insights into the dynamics of clonal diversity with the potential to develop sustainable solutions for future viticulture.

## Data Availability

Data sharing is not applicable to this article as no datasets were generated or analysed during the current study.
